# Implementation of a Radio-frequency Identification System to Improve the Documentation and Compliance of Attending Physicians’ Arrival to Trauma Activations

**DOI:** 10.7759/cureus.3582

**Published:** 2018-11-13

**Authors:** Sarah Stankiewicz, Rahul Kar, Aikaterini Hadoulis, Francesca Sullivan, William C Nugent, Jason Sample

**Affiliations:** 1 Surgery, New York-Presbyterian Queens, Flushing, USA

**Keywords:** radio-frequency identification, trauma activation, compliance, american college of surgeons

## Abstract

Background

The documentation of physician arrival is an important component of trauma resuscitation. The American College of Surgeons (ACS) requires attending physicians at Level I and Level II trauma centers to arrive to the most critical traumas, full trauma team activations (full activations), within 15 minutes at 80% compliance, and to limited trauma team activations (limited activations) within a timely manner, which we designated as 60 minutes. However, our institution’s rates of documentation and compliance using a paper-based trauma flowsheet (TFS) were found to be well below the 80% compliance rate.

Methods

Physicians began using a radio-frequency identification (RFID) badge to swipe into the emergency department (ED) upon arrival to the trauma room. Arrival times were taken from the swipes data and used to supplement missing or non-compliant times on the TFS. If a TFS was missing a time, it was considered both undocumented and noncompliant. A two-proportion z-test was used to compare the rates of documentation and compliance before and after the addition of swipes data.

Results

Documentation rates for full activations rose from 76% to 90%. Compliance rates for full activations rose from 70% (below the requirement) to 84% (compliant). Limited activation documentation and compliance rose significantly from 47.2% and 45.3% to 67.4% and 63.4%, respectively. Total documentation rose significantly from 49.9% to 69.7%. We went from below compliance to above compliance with the addition of the RFID system.

Conclusion

The use of the RFID technology improved the rates of documentation and compliance of attending physician arrival to trauma activations. Rates rose between 14 and 20 percentage points in each category, significantly in total documentation and in limited activation documentation and compliance. The addition of RFID swipes data made our rates improve to become compliant.

## Introduction

Documentation is an important component of trauma care in the emergency department (ED). Due to the large volume of data that needs to be recorded, a single nurse is typically designated to the role of scribing the events of the patient resuscitation during a trauma activation. Documentation by a scribing trauma nurse includes details of the assessment, intervention, and response time. A detailed and comprehensive report of events in the ED is an important strategy in the reduction of risk during patient encounters. The paper-based trauma flowsheet (TFS) is a multi-page documentation tool previously used by a majority of trauma centers, although the adoption of the electronic medical record (EMR) in recent years has reduced the number of paper-based TFSs in favor of electronic documentation. It contains designated areas for demographics, mechanism of injury, physician response time, and other vital measurements (Conference paper: Sarcevic A. Who's Scribing?: Documenting Patient Encounter During Trauma Resuscitation. ACM Conference on Human Factors in Computing Systems. 2010). In a fast-paced ED, it can become difficult to properly record the events taking place. The scribing nurse must document several events in the ED simultaneously, despite the confined and chaotic space. The scribing nurse may also be required to participate in the patient resuscitation, assisting the primary nurse with tasks such as drawing blood, delivering samples, and establishing intravenous access, taking their responsibility away from recording the patient encounter. Thus, many handwritten documentation methods contain both inaccurate and missing data (Conference paper: Sarcevic A. 2010).

An important byproduct of ED documentation is compliance with the standards of the American College of Surgeons (ACS). ACS standards require that attending surgeons at Level I and Level II trauma centers report to the ED for full trauma team activations (full activations) within 15 minutes of activation with an 80% compliance rate [1]. Individually, our institution requires that limited trauma team activations (limited activations) be reported to in 60 minutes or less. It is important to note that different institutions may have varying terms for the designated activation levels, such as level I or level II trauma activations, alpha or bravo activations, etc.

The radio-frequency identification (RFID) technology shows promise as a documentation tool given that it does not interfere with physician activities and is relatively simple to integrate into medical settings [2]. This technology involves the wireless storage and automatic retrieval of information. RFID systems are composed of two parts: tags and readers. The reader consists of one or more antennae that emit radio waves and receive information from the tag. The tag also emits radio waves that send stored information back to the reader [3]. The two most common forms of RFID systems are active and passive. Active systems involve tags with a built-in power supply, an active receiver, and an active transmitter. The reader can detect and read a tag from several meters away. Passive RFID systems have tags without an internal power source, and their range is significantly lower [4].

Passive RFID tags are often smaller and cheaper than active tags, which are generally larger and more expensive due to the active battery and increased memory inside. However, because of the internal battery, active tags have a much larger range and can pick up weaker signals. Passive tags require the use of the power source inside the reader to activate the tag, thus requiring very close proximity. Active tags are also more adept at working in areas with large amounts of metal, where the metal may interfere with signal reading for passive tags. Battery life, however, is a concern for active tags, as they cannot harvest power from the reader. Thus, passive tags have a longer lifespan than active tags [4].

RFID has previously been used in medical settings for various tasks, such as medical device and medication tracking. According to Wang et al., few hospitals have used the RFID technology to track patients and staff (Conference paper: Wang SW, Chen WH, Ong CS, Liu L, Chuang YW. RFID Applications in Hospitals: A Case Study on a Demonstration RFID Project in a Taiwan Hospital. 39th Hawaii International Conference on System Sciences. 2006). A recent literature search for RFID applications in the healthcare setting has yielded no peer-reviewed research articles, demonstrating the application of RFID for tracking staff within an institution. However, many RFID companies have advertised staff tracking as a function of their systems.

NewYork-Presbyterian/Queens (NYPQ) utilized the RFID technology to catalog attending physicians’ arrival to the ED, with the goal of improving documentation and compliance with ACS requirements. RFID capability is already built into the identification badges at NYPQ. A system is currently in place to allow badge access to restricted areas and to link medical professionals with tools or medications used in patient care. The application of the RFID system for tracking attending physicians required no additional implementation into everyday practices or technology. The RFID system at NYPQ is a passive system. There is a mounted RFID reader at each entrance to the designated trauma room accompanied by a reminder for staff members to swipe their badge when entering trauma activation. When attending physicians “swipe” into the ED, the RFID scanner reads the code on their badge and records the time of arrival. This data is then compiled in a database hosted by the Security department of NYPQ. The data is sent from Security to Trauma Services, where arrival times are matched with the appropriate trauma activation by the NYPQ trauma team staff. We wished to compare documentation and compliance rates under the previous TFS-only method to rates achieved with the addition of data from the RFID system.

## Materials and methods

This study examined full activations and limited activations from June 2016 through December 2016. For each activation, the TFS (paper copy or online scan) was examined for the attending physician’s name and time of arrival. The time of arrival was compared to the documented time of trauma activation to determine whether the attending physician arrived within 15 minutes of a full activation (ACS requirement) or 60 minutes of a limited activation (NYPQ standard). If either time of arrival or attending physician name was missing from the TFS, it was considered both noncompliant and undocumented.

Badge swipe data from the RFID system was then included to supplement the TFS results. For each activation, the badge swipe time stamp was compared with the documented time of trauma activation from the TFS to determine whether the attending physician arrived to the ED trauma area within the designated times for full and limited activations. After inclusion of the RFID data, rates of documentation and compliance were recalculated.

Rates were compared using a two-proportion z-test to determine whether there was a significant increase in compliance and documentation after the introduction of the swipes data. The data were analyzed in total and by activation level. The study was approved by the NYPQ Institutional Review Board.

## Results

Between June 2016 and December 2016, there were 531 trauma activations. Of these, 50 were full activations and 481 were limited activations. Total documentation before the introduction of the swipes data was 49.9% (n=265). Full activation documentation was recorded at 76% (n=38), with compliance recorded at 70% (n=35). Limited activation documentation and compliance were 47.2% (n=227) and 45.3% (n=218), respectively. After the inclusion of the swipes data, total documentation rose to 69.7% (n=370). Full activation documentation and compliance rose to 90% (n=45) and 84% (n=42), respectively. Limited activation documentation rose to 67.4% (n=324) and compliance rose to 63.4% (n=305). Using a two-proportion z-test, significance at p<0.05 was assessed. The difference in total documentation, limited activation documentation, and limited activation compliance was significant at p<0.0001. Full activation documentation and compliance were not significant at p=0.0624 and p=0.0962, respectively.

## Discussion

Documentation and compliance are a vital part of trauma activation. Poor documentation and compliance rates are common in a busy ED, making it necessary to relieve pressure on the scribing nurse by implementing additional methods of recording physician arrival time. The use of the RFID technology in the documentation and compliance of attending physician arrival times has shown to be successful at NYPQ. Total documentation increased 19.7 percentage points after the inclusion of the swipes data. Documentation and compliance for full activations both increased 14 percentage points, bringing compliance above the minimum rate required by ACS for full activations. Limited activation documentation increased 20.2 percentage points and compliance increased 18.1 percentage points. The use of the swipes data increased compliance and documentation in all categories and significantly for total documentation and limited activation compliance and documentation. Full activation compliance rose from below the required rate to above, proving that RFID can be used successfully to track staff arriving to the ED and act as a vital documentation tool for the maintenance of trauma center standards. The ease and accessibility of using a common, previously established form of technology make this approach to tracking attending physician arrival a valuable resource that many institutions can utilize.

While the addition of swipes data improved full activation documentation and compliance rates, the change was not statistically significant. NYPQ believes this is due to the high importance placed on compliance and documentation for these activations in comparison to limited activations. Not only are full activations the most serious and time-critical, but the ACS standard for compliance and documentation is the only formal requirement for trauma centers, as no other institution or verification organization exists that sets standards for physician arrival to trauma activations. NYPQ designates a 60-minute window for partial activations in accordance with the ACS mandate (found in the clarification document for Resources for Optimal Care of the Injured Patient section 5-16) that attending physicians respond to these activations in a timely manner [1]; however, there is no set standard or outside monitoring authority for this response. This sense of urgency, both for the patient and to meet ACS standards, likely contributes to the higher rates of compliance and documentation in full activations. Limited activations have a much longer response window than full activations. This may contribute to the significance of the RFID swipe additions for limited activations compared to the full activations. Because the window for arrival is larger, there may be a greater chance of the attending physician arriving during the activation without recognition by the scribing nurse. The larger response window may also contribute to the lower rates of compliance pre-implementation, as it increases the potential for attending physicians to miss the window if they feel they have time to prioritize other responsibilities. The addition of the RFID swipes may have influenced prioritization by supplying an additional and accurate monitoring source.

The use of passive RFID technology can be a disadvantage considering the increased benefits of using an active RFID system. Unlike the passive, the active tag can be read from further away and has its own internal power source. With the current system, the attending must mindfully swipe their badge on the reader. With an active system, simply walking into the ED trauma area would send the tag’s information to the reader and record the time of arrival. This would remove the possibility of human error in remembering to actively swipe the badge. System downtime and technological errors are also possibilities. It is important to note, however, that the use of the RFID system does not replace the use of the traditional TFS; rather, it acts as a safeguard to ensure compliance and accurate record keeping, supplementing the scribe nurses record on the TFS.

Additionally, there were some initial barriers to implementation, including physician resistance to swiping into the ED. Although Wang et al. describe user resistance as a reason for the failure of RFID in healthcare settings, swipes data from NYPQ have shown that despite initial user resistance, usage rates increased as the project progressed (Conference paper: Wang SW. 2006). It can be concluded that with the small patient population and trend towards acceptance of the swipe system, RFID technology use will increase. With increased awareness and proper training, it can be expected that the acceptance of swipe systems will increase.

## Conclusions

The fast pace of an ED can result in poor or missing documentation on a traditional TFS. To combat this, NYPQ introduced an RFID-based swipe system to track the attending physicians’ arrival to the ED. Despite initial resistance, documentation and compliance increased between 14 and 20 percentage points in each category, with significance in total documentation and limited activation documentation and compliance. The inclusion of the swipes data allowed compliance to reach the rate set by ACS standards. In conclusion, the introduction of an RFID swipe system was successful in increasing the compliance and documentation of trauma activations and reaching ACS and hospital standards.
